# Physiology

**Published:** 1836-07

**Authors:** 


### PHYSIOLOGY.

### *On the Power by which the Head of the Thigh-bone is retained in its Socket.* By Dr. Eduard Weber.

[At the meeting of German naturalists at Bonn, 25th September, 1835, a paper on this subject was read by Dr. Weber; and, on the following day, the experiments referred to in it were repeated before the Anatomico-Zoological Section of the Association. We shall give a translation of the greater part of this interesting paper, omitting merely the account of the experiments performed at the meeting, which were identical with those described in Dr. Weber's communication.]

It has been generally believed that it is through the power of the muscles or ligaments that the head of the thigh-bone is retained in its socket; and this, probably, because they are the first to strike our senses. A closer investigation, however, has proved that it is neither the muscles nor ligaments that effect this, but the pressure of the atmosphere. The head of the thigh-bone, enclosed in its air-tight cavity, remains in its place, on the same principle that the piston remains fixed in a cylinder, the upper extremity of which is closed with the finger; being forced by the air against the upper walls of its cavity, just as the mercury in the barometer is forced upwards by the same agent. The following experiments prove this fact:

1st. The trunk of the body was laid on a table in such a position that the leg and thigh hung down unobstructed. If the bone were supported in its place by the muscles and ligaments, it must clearly fall when the muscles and ligaments are divided. I divided the muscles and ligaments, and the limb did *not* fall: on the contrary, the bones constituting the joint remained in close contact, as before.

2d. If we admit that the limb is maintained in its position by atmospheric pressure, it is clear that it should fall down when air is admitted into the joint. I bored a hole through the wall of the bony cavity of the joint, and on the admission of the air the limb fell; and the displacement followed on boring the hole, even when the muscles and ligaments were not divided.

3d. If we assume that the atmospheric pressure is *alone* sufficient to maintain the limb in its place, the bone which had fallen down ought to be retained there when replaced and the air excluded. I replaced the bone which had been severed from the body, and excluded the air from the joint by closing the artificial opening with the finger: the limb remained supported in its place; and immediately fell down once more on the finger being removed.

I will only here refer to one instance of the practical application of this view of the mechanism of the thigh-joint,—the case known by the name of *spontaneous luxation of the thigh.* It is well known that, in this case, the bone is displaced without any obvious external cause, the limb being at first considerably lengthened, with marked lameness.

Since it has been shown that the head of the bone is not retained in its place by the power of the ligaments, it is not therefore necessary, for the explanation of the case, to admit that the ligaments must, previously to its occurrence, be lengthened, or (to speak generally) *altered,* in any way. Our experiments have shown that the head of the bone fell immediately from its socket into the inferior portion of the capsular ligament (in die höhle der kapsel herabsank,) upon the admission of air into the cavity of the joint. But, to produce this result, it is not necessary that air should be introduced from without: the same effect will follow the secretion of a fluid or the growth of a solid body in the cavity of the joint itself. In proportion as such a substance, whether fluid or solid, accumulates, the head of the bone will necessarily fall down through its own weight, without the application of any force, and without the least resistance being opposed by the ligaments of the joint.

[The experiments at the Association were witnessed, among others, by Leuckart, Mayer, Schulze, Munke, and the two Webers; all of whom were convinced of the retention of the bone in its place by the pressure of the atmosphere.]

*Müller's Archiv. für Anatomie, &c. Jahrgang,* 1836. Heft 1.

---

